# A discrepant presentation of bacteremia in the emergency department linked to a *Fusobacterium nucleatum* infection: a case report

**DOI:** 10.1186/s13256-021-03208-3

**Published:** 2022-01-04

**Authors:** Johnny Michel, Luc-Marie Joly, Virginie Eve Lvovschi

**Affiliations:** 1 Emergency Department, Rouen University Hospital, 76000 Rouen, France; 2 Normandie Univ, UNIROUEN, INSERM U 1073, CIC-CRB 1404, Rouen University Hospital, 76000 Rouen, France

**Keywords:** *Fusobacterium nucleatum*, Bacteremia, Emergency, Management, Blood culture, Febrile illness

## Abstract

**Background:**

*Fusobacterium nucleatum* is an anaerobic bacterium mainly responsible for acute or chronic infection of the ear, nose, and throat, potentially bacteremic with a risk of extraoral metastatic infection. Bacteremia occurs mainly in the elderly or in immunodeficient individuals, with high mortality. *F. nucleatum* is not the first cause of tonsillar infection in emergency departments, which are more often the consequence of a viral or streptococcal infection, but it is a risk factor for severe bacterial infection, especially in a viral pandemic context.

**Case presentation:**

A 25-year-old European woman with no history presented to the emergency department with fever (38.9 °C), pharyngeal symptoms, intermittent headaches, and alteration of general condition. On examination, she presented odynophagia associated with moderate tonsillar hypertrophy, her neck was painful but flexible. A rapid diagnostic test for beta-hemolytic group streptococcus was negative. First biological analyses revealed an inflammatory syndrome with C-reactive protein of 76 mg/L. Procalcitonin was measured secondarily, and was 2.16 µg/L. Faced with discordant clinical and biological findings, a lumbar puncture was performed, which came back negative. At hour eight, hypotension was observed but corrected after filling with physiological serum. The patient was hospitalized for monitoring, based on a hypothesis of severe viral presentation. At hour 24, pyrexia confirmed this hypothesis. A spontaneous but transient improvement and no new hemodynamic event led to early discharge. At day three, she was rehospitalized for increased and continuous headaches, without hemodynamic severity. A broad-spectrum probabilistic antibiotic therapy of ceftriaxone and metronidazole was started due to first blood cultures positive for anaerobic Gram-negative bacilli, while waiting for identification of the pathogen. Three days later, *F. nucleatum* was identified. According to the microbiological results, antibiotic therapy was adapted with amoxicillin and clavulanic acid, and no further complications were observed during clinical or complementary examinations. The final diagnosis was a *F. nucleatum* oropharyngeal infection complicated by bacteremia, without metastatic spread.

**Conclusion:**

The etiologies of tonsillar infection are not limited to benign viruses or bacteria. These should not be overlooked in emergency medicine, especially when the clinical presentation is discrepant. A combination of early bacterial investigations as blood culture and close clinical monitoring is the only safe way to detect bacteremia, especially in immunocompetent patients.

## Introduction

Currently, treatment strategies for oropharyngeal infections and outcomes in primary care [1] are based on recommendations linked to a high prevalence of streptococcus and viral agents. These are based on old epidemiological data [2].

*Fusobacterium nucleatum * is a commensal anaerobic bacterium present in the microbiota of the mouth and the digestive tract. The pathogenesis of *F. nucleatum* is linked to acute and chronic periodontal lesions, and may lead to intraabdominal, head, and neck infections and abscesses [3]. Moreover, at a systemic level, in cases of bacteremia, *F. nucleatum* is associated with invasive pulmonary and abdominal infections [4].

Here we present a low-symptomatic pharyngeal form of infection linked to *F. nucleatum* complicated with bacteremia, managed in the emergency department (ED), in a patient with no predisposing risk factors. Immunosuppressive treatment, sepsis, and radiation therapy can be differential diagnoses of unusual bacteremia [5].

In the literature, no data or clinical cases are available on nonspecific fusobacterium oropharyngeal presentations and their prognosis, especially in emergency medicine. This clinical case is a reminder that a diagnosis of bacterial infection should not be overlooked, even in a pandemic context such as Coronavirus-19 (COVID-19).

## Case presentation

A 25-year-old European woman with no history presented to the ED with a 24-hour fever associated with vomiting, diffuse aches and pain, and intermittent headaches. The patient had no recent travel. On examination, her temperature was 38.9 °C, blood pressure was 109/72 mmHg, and heart rate was 104 beats/minute. At ear nose and throat (ENT) examination, the patient presented odynophagia associated with moderate tonsillar hypertrophy without cervical adenopathy; her dental status was good with no gingivitis; her neck was painful but flexible. The rest of the clinical examination was normal. A rapid diagnostic test for beta-hemolytic group streptococcus was negative. First biological analyses revealed an inflammatory syndrome with C-reactive Protein (CRP) 76 mg/L, hyperleukocytosis (16.6 g/L), and neutrophils 14.7 g/L. Protidemia was 77 g/L and metabolic acidosis was suspected with a bicarbonate level of 15 mmol/L and an anion gap of 20 mmol/L, indicating extracellular dehydration. Urine test strip was negative. Chest X-ray was normal. Blood cultures were grown. Procalcitonin (PCT) was measured secondarily, and was 2.16 µg/L. Faced with discordant clinical and biological findings (a low-symptomatic form of ENT infection with high PCT), a lumbar puncture was performed, which came back negative (Table 1).

**Table 1:** Microbiological analyses during hospitalization

Cerebrospinal fluid analysis		Blood cultures		Urinary analysis	
Appearance	Clear	Day 1/Baseline	Day 3	Day 1/Baseline	Day 3
Leukocytes	< 2/mm^3^	Aerobic flask: negative Anaerobic flask: Gram negative bacilli and identification of *Fusobacterium nucleatum*	Aerobic flask: negative Anaerobic flask: negative	Leukocytes: 10^4^–5 × 10^4^/mL Culture: negative	Leukocytes: 5 × 10^4^–10^5^/mL Culture: negative
Red blood cells	< 2/mm^3^				
Protein (g/L)	0.28				
Glucose	0.6 g/L or 3.6 mmol/L				
Chloride (mmol/L)	125				
Lactate (mmol/L)	1.88				
Culture	Negative				

**Fig. 1:**
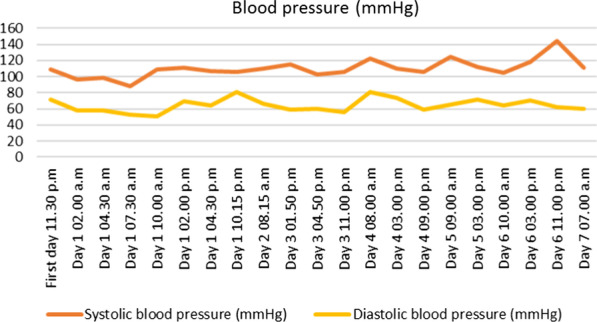
Evolution of blood pressure

**Fig. 2:**
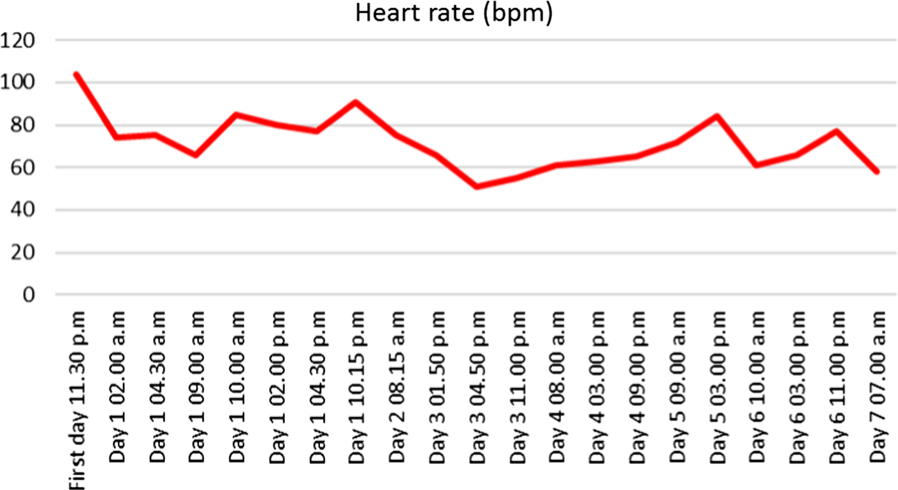
Evolution of heart rate

**Fig. 3:**
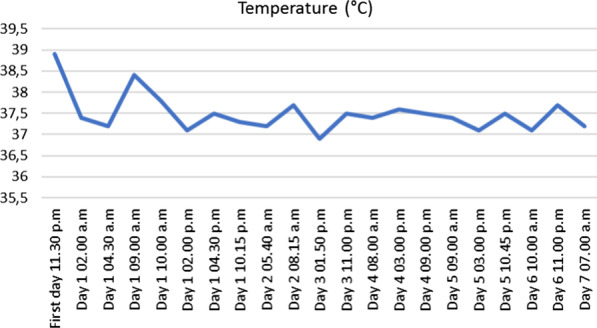
Evolution of temperature

**Fig. 4:**
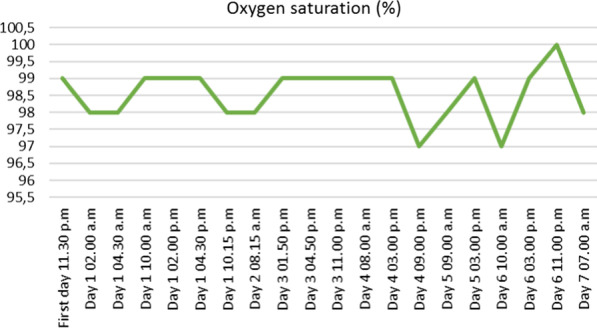
Evolution of pulsed oxygen saturation

**Fig. 5:**
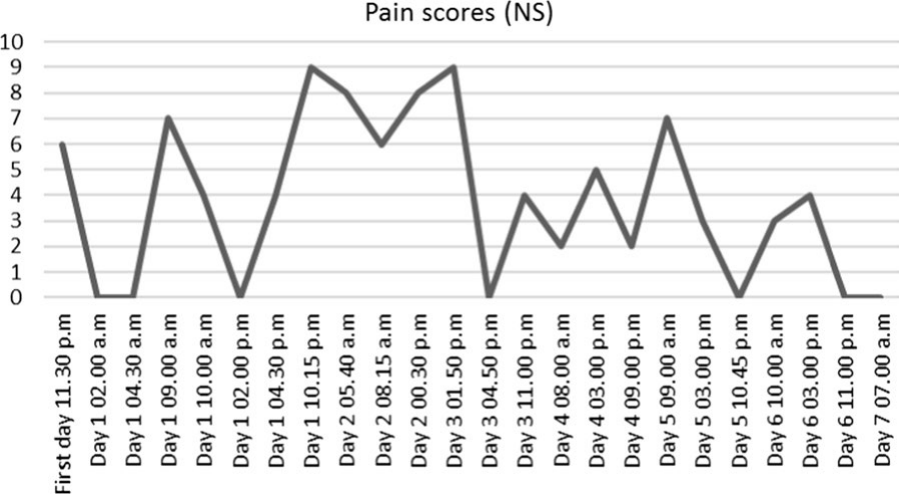
Evolution of pain score using a numeric scale (NS)

At hour eight, hypotension was observed (88/53 mmHg) but corrected after filling with 500 mL of physiological serum. The patient was hospitalized for monitoring, based on the hypothesis of a severe viral presentation, associated with metabolic acidosis and elevated biomarkers (Table 2). At hour 24, spontaneous pyrexia occurred, confirming this hypothesis. CRP was stable and no new hemodynamic event was observed (Figures 1, 2, 3, 4 and 5). The patient was discharged home.

**Table 2:** Biological analyses during hospitalization

Day	Day 1/baseline	Day 2	Day 3	Day 4	Day 5
Red blood cells (t/L)	4.49	4.28	4.54	4.16	4.21
Hemoglobin (g/dL)	13.6	12.5	13.6	12.5	12.5
Hematocrit	0.39	0.38	0.41	0.37	0.38
Leukocytes (giga/L)	16.6*	10.0	9.1	6.9	6.9
Polynuclear neutrophils (giga/L)	14.72*	6.56	6.28	3.63	**3.02**
Lymphocytes (giga/L)	1.06*	2.13	1.82	2.17	2.70
Monocytes (giga/L)	0.78	0.84	0.50	0.46	0.50
Platelets (giga/L)	284	242	268	245	271
Prothrombin time (%)	83				
Activated partial thromboplastin time (seconds)	1.12				
Total bilirubin (mmol/L)	11			5	3
Alkaline phosphatase (UI/L)	57			45	47
Gamma GT (UI/L)	12			14	17
Aspartate aminotransferase (UI/L)	25			33	39
Alanine aminotransferase (UI/L)	18			39*	36
Urea (mmol/L)	2.2*	3.1	2.5	2.8	2.0*
Serum creatinine (mmol/L)	61	61	56	61	58
Sodium (mmol/L)	138	140	141	140	140
Potassium (mmol/L)	3.5*	3.9	4.1	4.1	4.1
Chloride (mmol/L)	104	107*	*108	106	107*
Alkaline (mmol/L)	15*	21*	17*	23	20*
Protein (g/L)	77	69	74	65	65*
C-reactive protein (mg/L)	76*	69*	24*	16*	10*
Procalcitonin (µg/L)	2.16*				
hCG (UI/L)	< 5				

*Results out of range

*T/L* teta per litre, *giga/L* giga per litre, *g/dL* gram per decilitre, *g/L* gram per litre, *mg/L* milligram per litre, *mmol/L* millimolar per litre, *GT* glutamyltranspeptidase, *UI/L* international unit per litre

The patient returned to the ED on day three, with increased and continuous headaches, chills and aches, but no hemodynamic severity. Two hours after arriving in ED, first blood cultures came back positive for anaerobic Gram-negative bacilli, while waiting for complete identification of the pathogen. Therefore, a probabilistic antibiotic therapy with intravenous ceftriaxone (1 g/24 hours) and oral metronidazole (500 mg/8 hours) was started and the patient was hospitalized for 4 days. Three days later, additional blood cultures identified*F. nucleatum* and the first antibiotic therapy was switched to an oral combination of amoxicillin (2 g/day) and clavulanic acid (125 mg/day) on day four for 10 days. Jugular venous thrombosis was searched by Doppler examination and found negative. Human immunodeficiency virus (HIV), hepatitis B virus (HBV), hepatitis C virus (HCV), cytomegalovirus (CMV), and syphilis serologies were negative, eliminating alternative diagnoses and immunodepression factors. Epstein–Barr virus (EBV) serology showed an old immunization profile. The final diagnosis was an *F. nucleatum* oropharyngeal infection complicated by bacteremia without metastatic spread.

## Discussion

Anaerobic oropharyngeal infections are rare, and the most frequent pathogen involved is *Fusobacterium necrophorum*, leading to Vincent’s angina or acute necrotizing ulcerative gingivitis [6] and, to a lesser extent, *F. nucleatum* and other Fusobacteria. In young patients, Vincent’s angina is responsible for Lemierre’s syndrome, a known locoregional complication of upper airway infections caused by thrombophlebitis of the jugular vein. In addition, *Fusobacterium* sp. are responsible for 1% of bacteremia of all origins, while 44% of bacteremia are related to anaerobic bacteria [7]. The most common species are *F. nucleatum* (60–70% of cases) and *F. necrophorum* (25–30%). *F. nucleatum* is less common in anaerobic oropharyngeal infections, but it must not be overlooked in the ED because it is frequently complicated by bacteremia [4].

Low-symptomatic pharyngeal forms of *F. nucleatum* infections, contrary to typical ENT clinical presentations of Vincent’s angina much better known by ED practitioners [6], can complicate and delay the diagnostic process. This particular presentation can lead to delayed diagnosis and increased risk of systemic complications and extraoral metastatic infections (brain, liver, joints, heart). The mortality rate associated with *Fusobacterium *sp. bacteremia varies from 5% to 47% [8]. In the majority of cases, bacteremia occurs in older individuals, and immunodeficiency is one of the risk factors that must be investigated [7].

The literature on the management in the ED of these ENT infections with a risk of bacteremia is rare. Numerous studies have focused on the high prevalence of *F. nucleatum* in colorectal cancer and its potential severity in digestive disease [9].

The present clinical case highlights the following: first, the need to be aware of major complications in cases of ENT infection without identified tonsillar infection, and second, the need to consider the possibility that these are not linked to classical aerobic bacteria as viral agents. Consequently, we should question our use of diagnostic methods for oropharyngeal infection and our antibiotic prescription decision in the ED: oropharyngeal infection associated with *F. nucleatum* requires a different antibiotic treatment [4] to that associated with group A streptococcus, the most frequent bacterial etiology (20%) [2] targeted by current management algorithms [1]. Fusobacterium species are naturally resistant to macrolides, quinolones, aminoglycosides, and trimethoprim molecules. Moreover, for *F. nucleatum*, resistance to penicillin through the production of penicillinases is increasing (30% in Europe) [10]. According to the prevalence of microbiological profiles in the general population, the common error is probably to consider a common oropharyngeal infection with a negative rapid diagnostic test for beta-hemolytic group streptococcus as a viral infection. The diagnostic process must follow an approach such as in urinary tract infections, considering negative rapid strip test. This is even more relevant in the current context, in which infection with COVID-19 is mainly sought when faced with an acute undifferentiated fever. Even though ENT manifestations in COVID-19 are less common than fever and cough, they can reach 44% in some studies [11]. The clinical reasoning must systematically integrate the search for a bacterial infection and, in particular, carry out a blood culture when considered necessary.

This clinical case shows that low-symptomatic forms of ENT infection with alteration of general condition should be considered by analogy with febrile illness entity [12], an important issue in emergency medicine: the final diagnosis of bacterial infection may not be complete, while systemic repercussions are kinetically at the forefront with a delayed initial diagnosis.

**Fig. 6:**
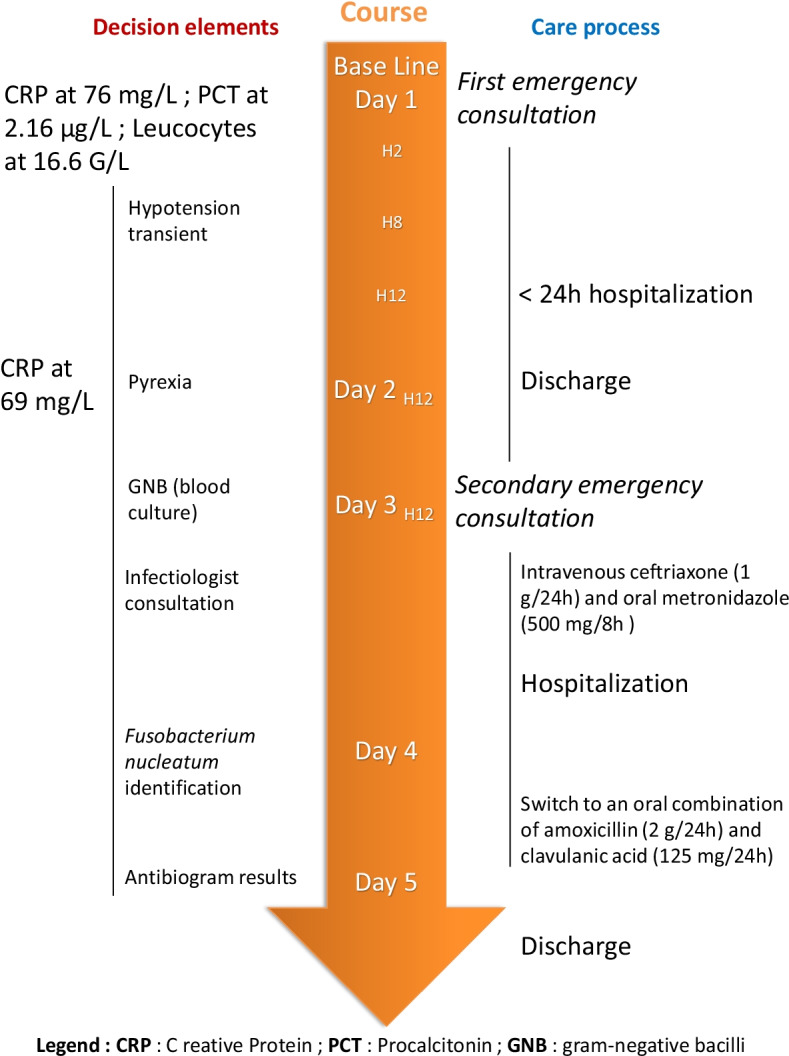
Complete management from presentation to discharge: treatments, outcomes

In this clinical case, a first step was facilitated by elevated serum biomarkers of inflammation, regularly implicated in severe bacterial infection [13], and initial 36-hour hospital monitoring was decided. Clinical presentation could have led to the conclusion of a severe form of “common seasonal virosis” with transient poor hemodynamic tolerance, indicating hypovolemia by dehydration. However, biological elements associating hyperleukocytosis, elevated CRP, and PCT levels, alerted us to the possibility of a systemic bacterial infection. The use of biomarkers remains an aid in the initial phase of management (Figure 6), but does not always allow conclusions of bacterial origin.

In a second step, decreased levels of inflammatory biomarkers, associated with pyrexia, led to hospital discharge without indication for probabilistic antibiotic coverage [14]. Few studies on CRP kinetics are available to guide intrahospital management of bacterial [15] or viral infections, especially in the ED. An elevated CRP level despite antibiotic therapy is known to be useful to suspect a fluid collection, but its decrease has been poorly studied [14]. Thus, it is difficult to evaluate the relevance of the initial phase of management without antibiotic coverage.

Finally, it was the complete bacteriological diagnosis of *F. nucleatum* bacteremia in a third and last step that allowed a targeted etiological treatment, after a delay of 3 days, linked to a long incubation period of the Fusobacterium genus (72–96 hours of anaerobic culture).

Fortunately, a first-reasoned probabilistic treatment was administered as soon as a Gram-negative anaerobic pathogen was identified on blood culture, 24 hours before the final diagnosis, and 48 hours after the ED presentation. Moreover, carrying out blood culture is an approach with a low impact on organizational constraints and a positive medicoeconomic balance, regarding the objective of preventing septic shock.

## Conclusions

A diagnosis of nonstreptococcal bacterial oropharyngeal infection should not be overlooked in emergency medicine, especially when the clinical presentation is discrepant: nonspecific pharyngeal manifestations associated with septic presentation suggested by biological results. The early use of inflammatory biomarkers remains useful for initial management but can be confusing in case of a long diagnostic process, leading to a delay in introducing antibiotic therapy. A combination of early bacterial investigations as blood culture and close clinical monitoring is the most relevant strategy to detect bacteremia and to avoid erroneous hypotheses of virosis faced with pharyngeal symptomatology and alteration of general condition, particularly in a viral pandemic context. An intrahospital management of infections with systemic effects, even transient, can be safe while waiting for final microbiological analyses.

## Data Availability

Not applicable.
